# A comparison of misoprostol with and without methylergometrine and oxytocin in outpatient medical abortion: a phase III randomized controlled trial

**DOI:** 10.1186/s13104-023-06509-6

**Published:** 2023-10-05

**Authors:** Athar Rasekh Jahromi, Vahid Rahmanian, Hajar Taghizadeh, Zahra Zareibabaarabi

**Affiliations:** 1 Obstetrics and Gynecologist, Departments of Obstetrics and Gynecology, Jahrom University of Medical Sciences, Jahrom, Iran; 2 Department of Public Health, Torbat Jam Faculty of Medical Sciences, Torbat Jam, Iran; 3 Research Center for Noncommunicable Diseases, Jahrom University of Medical Sciences, Jahrom, Iran

**Keywords:** Ambulatory care, Miscarriage, First Trimester, Clinical trial

## Abstract

**Objective:**

The complications associated with miscarriages have surfaced as a major concern in maintaining women’s physical and mental health. The present study evaluated the efficacy of three medication regimes for the complete expulsion of retained intrauterine tissues in patients who underwent a miscarriage.

**Methods:**

In this randomized clinical trial, 90 patients participated with their gestational age below 12 weeks, each having undergone a recent miscarriage. After being screened for underlying diseases and coagulative blood disorders, they were randomly allocated into three groups. For the first group, labeled as the control group, misoprostol was administered alone. In contrast, the combination of misoprostol plus methylergometrine and misoprostol plus oxytocin was prescribed for the second and third groups, respectively. Further, the data obtained were analyzed by descriptive and inferential statistics using Stata software version 14.

**Results:**

The mean age of participants and gestational age were 29.76 ± 5.53 years and 8.23 ± 2.29 weeks, respectively. There was no significant difference between the three treatment groups regarding the amount of bleeding after the abortion(P = 0.627). Regarding pain severity, the group that received Misoprostol plus Methylergometrine had less pain intensity than the other two groups(p = 0.004). The mean rate of RPOC expulsion was in the Misoprostol plus Oxytocin (9.68 ± 10.36) group, Misoprostol plus Methylergometrine (11.73 ± 12.86), and Misoprostol groups (19.07 ± 14.31)(p = 0.013). The success rate in outpatient medical abortion in the misoprostol plus oxytocin and misoprostol plus methylergonovine group was 93.33%, but in patients treated by misoprostol alone was 83.33%.

**Conclusion:**

The effectiveness of the drugs in the two drug groups combined with oxytocin and methylergometrine is higher than the misoprostol group alone. An outpatient approach was deemed more satisfactory against surgical maneuvers and hospitalizations by patients since family support influenced their pain coping mechanism.

**Trial registration:**

The trial was registered in the Iranian registry of clinical trials on 04/10/2019. (https://fa.irct.ir/trial/34519; registration number: IRCT20150407021653N19).

## Introduction

Globally, the complications succeeding a spontaneous or unsafe abortion have been recognized as an impediment to women’s general well-being [1]. A systematic and analytical study from the World Health Organization (WHO) estimated a mortality rate of 7.9% in mothers who take on unsafe or spontaneous abortion [2]. Another study from 2017 revealed that 18% of all pregnancies in the USA were aborted, with 80% being related to a gestational age of fewer than ten weeks [3].

The prevalent long-term complications of intrauterine tissue retention include infertility, bleeding, intrauterine adhesion, coagulation disorders, infection, and death, which are in a linear relationship with age advancement [4]. In contrast to curettage and manual vacuum aspiration, medical therapy by misoprostol, also known as prostaglandin, is considered a safe, non-invasive, and effective alternative to managing intrauterine fetal death [5–7]. A study by Ibiyemi et al. illustrated that 600 micrograms of oral misoprostol intake could completely expel retained tissue [8].

Some studies have been carried out to ascertain the expulsion rate of persistent placental or trophoblastic tissue, to compare oxytocin efficacy on uterine smooth muscle contractions, and also to relate the prophylactic effects of methylergometrine on postpartum hemorrhage [9–12]. These studies have compared different drug choices, such as single-drug administration or combined medical therapy during hospitalization. However, no record of outpatient treatment for residual tissue expulsion in patients with first-trimester incomplete abortion was found.

This study aimed to investigate the efficacy and safety of misoprostol for treating retained products of conception (RPOC) following a miscarriage, with the added importance of avoiding hospitalization during the COVID-19 pandemic. To evaluate the efficacy and side effects of each medication regime, a comparison was drawn between the administration of misoprostol with the combined use of misoprostol plus oxytocin and misoprostol plus methylergometrine for complete expulsion of retained intrauterine tissues in patients who underwent a miscarriage. The outcome evaluated whether outpatient treatment could be substituted with a surgical approach as a safe maneuver.

## Main text

### Methods and materials

#### Study design

This clinical trial was performed on 90 patients referred to the gynecology and obstetrics clinic affiliated with Jahrom University of Medical Sciences with miscarriage and a gestational age below 12 weeks from March to July 2020.

The sample size was calculated according to Paris et al. [13], considering the 0.8 effect size, type 1 error (α) 5%, power 80%, and considering the non-response rate of 10%, the required sample size of 90 people (30 people for each group), Furthermore, we did power analysis based on information from the previous study [13] using the G*Power software by type of A priori method [14, 15] and Actual power (1-β err prob) was calculated” as 0.8074866.

After taking a comprehensive history from each patient, the data obtained was registered in the relevant code sheets. After being matched in gestational age, the next step involved randomly allocating patients into three distinctive groups. To reduce outcome assessment bias in this study, the radiologist examined the images without prior knowledge of the patient’s status, thus ensuring impartial evaluation. Subsequently, the patients were checked for success rates of pregnancy tissue expulsion as the primary outcome and bleeding, pain, and spotting as a secondary outcome. The protocol of this study was approved by the ethical committee of Jahrom University of Medical Sciences issued with code No.: IR.JUMS.REC.1396.143 was further registered in the Iranian registry of clinical trials with code ID IRCT20200122046221N1. It is worth mentioning that all patients recruited in this study have provided their written informed consent to the researchers, and all probable adverse events and benefits of the research were explained to them.

#### Participants

The eligibility criteria assigned for the patients to enter the study were as follows: gestational period below 12 weeks as confirmed by sonography & Last Menstrual Period (LMP); spontaneous and/or incomplete abortion diagnosed by sonography; and vaginal bleeding history in the current pregnancy that encouraged patients to pursue outpatient treatment to expel RPOC. However, the exclusion criteria that disqualified the patients for the study were the following: hypersensitivity to misoprostol or certain prostaglandins; drug limitations for taking prostaglandins in patients with asthma, glaucoma and hypertension, hepatic disorders, seizure history, history and/or presence of thromboembolism; smoking habits; Intrauterine device (IUD); Hb < 10 mg/dl; temperature > 38º C and finally, pelvic infection or sepsis that caused patient’s discontentment for outpatient treatment. Moreover, an unstable hemodynamic status was considered among the exclusion criteria.

### Randomization and blindness

Random allocation was done using Random Allocation Software as a random division with the nine-way random block method by an epidemiologist who was not involved in the study. Using the lottery, the letters A, B, and C will be considered symbols of one of the three intervention groups. In the next step, each of the nine codes was placed in a sealed envelope that could not be read from the envelope. At the patients’ visit, one of the envelopes was randomly selected, and the patients were assigned to one of the study arms in the order of the letters mentioned.

In this study, it was impossible to blind the participants or researchers to the treatment group assignments, as each group received a different medication regimen, and participants were aware of the treatment they were receiving. Additionally, this study involved outpatient treatment, and participants had to go to the nearest clinic to receive injections, reducing the likelihood of blinding.

#### Intervention

A thorough and organized history was obtained from the patients who met the criteria to participate in the study. Additionally, they received a particular informed consent form to acquaint them with the research objectives and were categorized into three groups regarding gestational age. The first group of patients was designated to be the control group. It was given misoprostol 200 mg (Misoglandin, Samisaz Co, Mashhad, Iran) every 6 h sublingually and three suppositories through the posterior vaginal fornix per night [16, 17].

The second and third groups followed different medication regimes as their treatment. In the second group, misoprostol 200 mg was prescribed in the form of sublingual tablet intake every 6 h and three suppositories through the posterior vaginal fornix combined with intramuscular methylergometrine 0.2 mg (Minoo Co, Tehran, Iran) thrice a day. These groups were also advised to refer to the closest clinic to take the injection cautiously [17, 18].

On the other hand, the third group was prescribed misoprostol 200 mg sublingually every 6 h and three suppositories through the posterior vaginal fornix every night. Also, this group was simultaneously administered oxytocin (Vetocin, Aburaihan Pharma Co., Tehran, Iran), 30 units in the morning and 30 units intramuscularly in the afternoon. It was advised to refer to the nearest healthcare center for these injections [16].

Likewise, in those patients with negative Rh, an intramuscular Rhogam 300 mg injection was prescribed to prevent iso-immunization [17, 18]. So, all the patients received treatment in the outpatient setting. After a 3-day interval, sonography was performed on each group to check the size of residual mass to assess the success rate of the undergoing outpatient treatment. The sonography was performed by an expert radiologist blinded to the patient’s therapeutic regime. Moreover, the course of the medication regime was repeated three times in the case of sonographic detection of endometrial mass. At last, a failure of the study was ascertained even with a slight detection of tissue residue under sonography, and patients with medical treatment failure were then advised to undergo other approaches, such as surgery or curettage, to remove the RPOC.

Participants in all three study arms received education on potential side effects to ensure the safe use of oxytocin and other regimes in the outpatient setting. They were instructed to report any adverse events promptly to the gynecology and obstetrics clinic affiliated with Jahrom University of Medical Sciences. The clinic was well-equipped to handle potential complications, and participants were fully monitored throughout the study. These safety measures were implemented to minimize the risks associated with outpatient use of oxytocin.

#### Data Collection

A demographic questionnaire used to gather information about age, sex, weight, blood pressure, existing underlying diseases, and the gestational age of the patients was given to the patients to fill in. Also, sonographic studies were employed to confirm intrauterine death.

After a 3-day outpatient intervention, the patients were asked to refer to the gynecology and obstetrics clinic affiliated with Jahrom University of Medical Sciences for further follow-up. In this follow-up session, data such as spotting in pregnancy, pain, bleeding, and finally, successful expulsion of RPOC were asked and recorded carefully in relevant code sheets. For the assessment of the pain severity in the patients, the Visual Analogue Scale system (VAS), a 10 cm scaled ruler, was used to show the degree of the pain in these patients, such that 0 and 10 were its two extremes representing no pain and worst pain, respectively. Data during follow-up were recorded in a blinded fashion and using forms prepared in advance. Furthermore, a 1–3 score denoted relative pain, 4–6 moderate pain, and 7–10 severe pain overall [19]. Other studies have confirmed the reliability and validity of the VAS scale, and Cronbach’s alpha value has been reported as 0.88 [20–23].

### Statistical analysis

Data analysis was done using Stata software version 14. At first, the Kolmogorov-Smirnov test was used to check the normality of the data. The descriptive statistics section reported the results as mean, standard deviation, frequency, and percentage. The analytical statistics section used one-way ANOVA, Chi-square, and independent-sample t-test. Furthermore, the Significance level for all tests was less than 0.05.

## Results

### Population characteristics

**Fig. 1:**
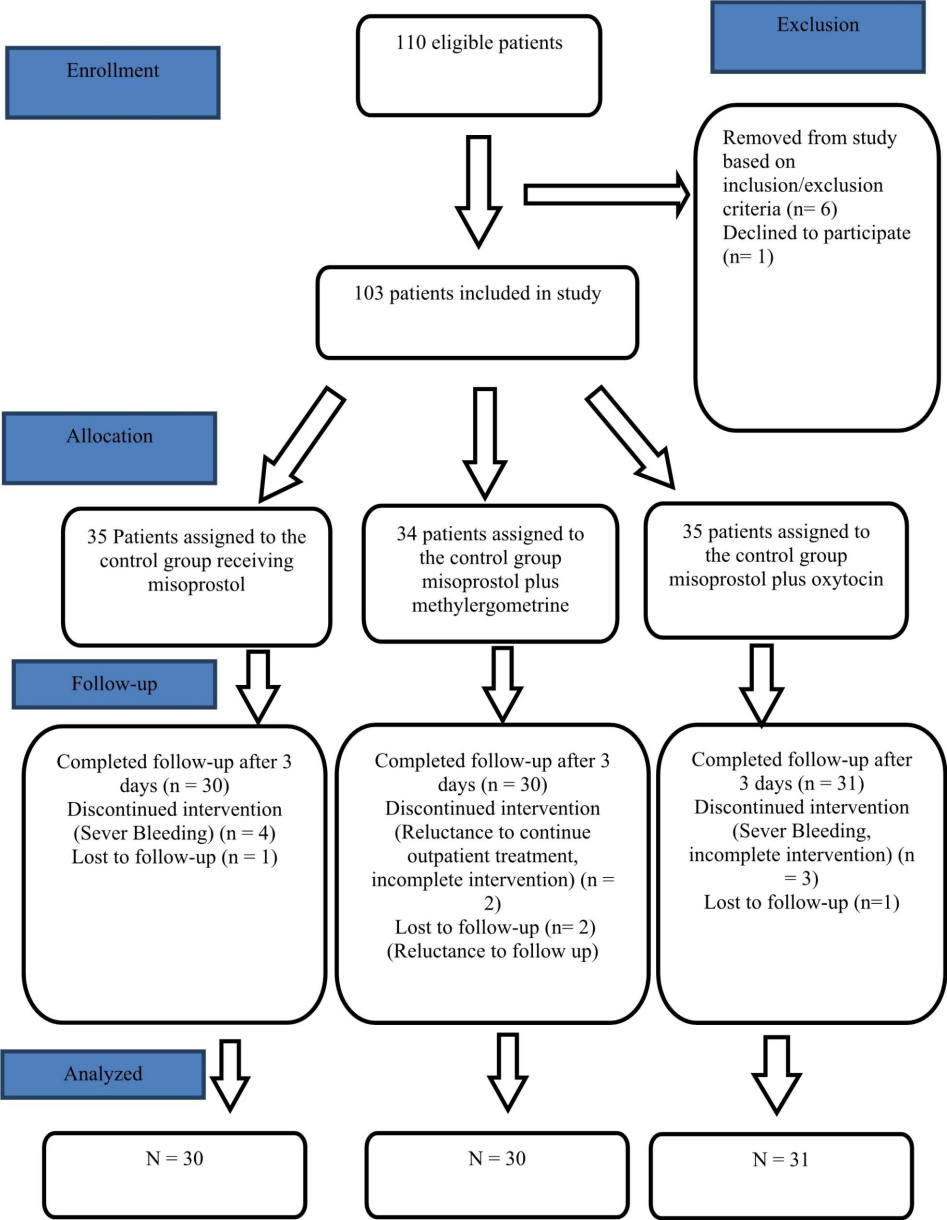
The CONSORT flow chart of the randomized trial regarding administered drugs in abortion

In this study, among the 110 eligible patients, 90 patients were evaluated and randomly allocated into three distinctive groups, each consisting of 30 participants. The first, second, and third groups received a dose of misoprostol, misoprostol plus methylergometrine, and misoprostol plus oxytocin, respectively, and were thus named accordingly(Fig. 1).

The mean age of all the patients and the mean gestational age attained 29.76 ± 5.53 and 8.23 ± 2.29, respectively. The weight, height, age, and blood pressure variables in the intervention and control groups were not significantly different (p > 0.05, Table 1).

**Table 1:** Comparison of demographic variables in three groups under study before intervention

Variable	Total	Groups			P-value*
		Misoprostol	Misoprostol + Methylergometrine	Misoprostol + Oxytocin	
*Age, yr*	29.76 ± 5.53	31.47 ± 5.83	28.13 ± 4.8	29.67 ± 5.57	0.064
*Weight (Kg)*	66.27 ± 11.07	64.59 ± 9.69	65.71 ± 10.61	68.48 ± 12.7	0.391
*Height (CM)*	159.98 ± 5.24	159.79 ± 4.88	161.22 ± 5.69	158.96 ± 5.07	0.275
*Min. BP (mm Hg)*	74.24 ± 9.84	74.6 ± 10.83	73.96 ± 10.12	74.17 ± 9	0.973
*Max. BP (mm Hg)*	116.6 ± 11.85	117.92 ± 9.25	118.96 ± 15.54	113.31 ± 9.56	0.167
*Gestational Age(Week)*	8.23 ± 2.29	8.17 ± 2.77	7.43 ± 1.59	9.14 ± 2.22	0.425

*One-way ANOVA, significance level < 0.05

### Comparison of therapeutic effects

There was no significant difference between the three treatment groups regarding the amount of bleeding after the abortion(P = 0.627). Still, in the Misoprostol + Methylergometrine group, 80% of the patients were in the mild bleeding group. In contrast, in the two groups of Misoprostol + Oxytocin and Misoprostol alone, this amount was 63.33% and 53.33%, respectively(Table 2).

**Fig. 2:**
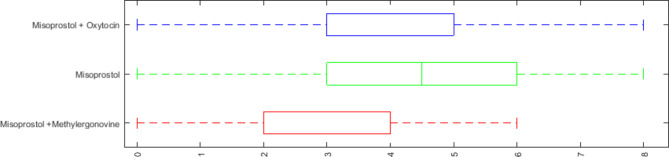
Minimum, maximum, and median of pain severity based on VAS scores in three groups under study

In terms of pain severity, there was a statistically significant difference between the three groups(p = 0.004). The group receiving Misoprostol + Methylergometrine had less pain intensity than the other two groups (Table 2; Fig. 2).

In addition, no statistically significant difference was observed between the three groups regarding spotting after abortion (P = 0.894) (Table 2).

**Table 2:** Abortion indications in three groups under study

Abortion indications		Groups			P-value*
		Misoprostol *n = 30*	Misoprostol + Methylergometrine *n = 30*	Misoprostol + Oxytocin *n = 30*	
**Bleeding**	*Mild*	16 (53.33(	24 (80)	19(63.33)	0.627
	*Moderate*	5 (16.67%)	1 (3.33)	4(13.33)	
	*Severe*	1 (3.33)	0 (0)	1(3.33 )	
	*No bleeding*	8(26.67)	5(16.67)	6(20)	
**Pain**	*None to Mild(VAS = 0–3)*	13 (43.33)	20(66.66)	17 (56.66)	0.004
	*Moderate* *(VAS = 4–6)*	11(36.66)	10(33.33 )	8(26.66 )	
	*Severe* *(VAS = 7–10)*	6(20)	0( 0)	5(16.66)	
**Spotting**	Yes	3 (10)	3 (10)	4 (13.3)	0.894
	No	27(90)	27(90)	26(86.7)	

*VAS: Visual Analogue Scale, * Chi-square test, significance level < 0.05

Assessing the rate of RPOC expulsion, no significant difference was seen before the intake of different medications(P = 0.434). However, a statistical difference was detected after patients’ exposure to these medications(P = 0.013). The mean rate of RPOC expulsion was in the Misoprostol + Oxytocin(9.68 ± 10.36) group, Misoprostol + Methylergometrine (11.73 ± 12.86), and Misoprostol groups (19.07 ± 14.31). As can be seen, the effectiveness of the drugs in the two drug groups combined with oxytocin and methylergometrine is higher compared to the misoprostol group alone (Table 3).

Finally, the outcomes demonstrated an 83.33% success rate in outpatient medical abortion patients treated with misoprostol alone. In contrast, those groups treated with misoprostol plus oxytocin and misoprostol plus methylergonovine illustrated a success rate of 93.33%. In addition, the results showed no statistically significant difference in drug side effects in the treatment groups under study (P = 0.329) (Table 3).

**Table 3:** Comparison of retained products of conception (RPOC) and state of ovary before and after medical therapy in three groups under study

Variable	Total	Groups			
		Misoprostol	Misoprostol + Methylergometrine	Misoprostol + Oxytocin	P-value
Degree of RPOC before medication, mean ± SD	32.49 ± 20.38	35.38 ± 17.22	28.55 ± 20.63	33.26 ± 23.01	0.434*
Degree of RPOC after medication, mean ± SD	13.53 ± 13.13	19.07 ± 14.31	11.73 ± 12.86	9.68 ± 10.36	0.013*
P-value** *	< 0.001	< 0.001	< 0.001	< 0.001	NA
The state of the ovary before medication, Frequency(percent)	normal	20(66.7)	20(66.7)	20(66.7)	0.355* ** *
	anormal	1(3.3)	1(3.3)	0(0)	
	cyst	6(20)	5(16.7)	4(13.3)	
	low few	2(6.7)	0(0)	0(0)	
	PCOS	1(3.3)	4(13.3)	6(20)	
The state of the ovary after medication, Frequency(percent)	Normal	23(76.7)	21(70)	18(60)	0.237* * *
	Cyst	2(6.7)	4(13.3)	2(6.7)	
	Low few	3(10)	0(0)	2(6.7)	
	PCOS	2(6.7)	5(16.7)	8(26.7)	
Complete abortion, Frequency(percent)	Yes	25(83.3)	28(93.3)	28(93.3)	0.329* * *
	No	5(16.7)	2(6.7)	2(6.7)	

RPOC: Retained products of conception, PCOS: Polycystic ovary syndrome, *One-way ANOVA, * * independent t-test,* * * Chi-square test NA: Not applicable, significance level < 0.05

## Discussion

The result of this study confirms an improved efficacy of the medical therapy of misoprostol, either combined with oxytocin or with methylergonovine, in successful expulsion of the RPOC. Although treatment by misoprostol alone (83.3%) has not resulted in remarkable statistical significance, greater therapeutic efficacy was reported in the groups (93.33%) treated by a combination of misoprostol plus oxytocin or misoprostol plus methylergonovine. Moreover, by designating the degree of persistent residual tissues in the patients, it was concluded that those treated with misoprostol plus oxytocin had the best outcome compared to the other groups. Likewise, all the patients treated with other medications showed a sufficient successful rate of RPOC expulsion.

Kaviani et al. concluded that ‘the efficacy of methylergonovine in the successful expulsion of retained pregnancy tissues in patients with incomplete abortion showed 94.6%’ [12]. Additionally, Niroumanesh et al. [24] reported results similar to our findings. Another study was carried out by Petco et al. [25] compared the effectiveness of simultaneous administration of misoprostol and oxytocin (M&O) versus misoprostol and mifepristone (M&M) on abortion in the second trimester in over 108 patients. Their study further illustrated that administering M&M simultaneously resulted in greater expulsion rates within 12 hours compared to M&O, with the latter regimen demonstrating synergistic benefits, lower expenses, and satisfactory I-AI.

Misoprostol is a prostaglandin E1 analog, a uterotonic pharmacological agent that functions either by contracting the uterine smooth muscles or by dilating the uterine cervix [26]. On the other hand, by increasing the permeability of uterine myofibrils to sodium, oxytocin causes uterine smooth muscle contraction. This compound is frequently used to induce labor [27]. Overall, these beneficiary medications aid in dilating the cervix gently, resulting in a drastic reduction in the subsequent complications of cervical dilation, i.e., uterus perforation, cervix rupture, hemorrhage, incomplete fetal-placental delivery and infection, and pregnancy product expulsion. Besides, these medications help to avoid anesthesia and its complications in cases where complete abortion is necessary [28].

Although the mean age of all the patients in this study was 29.76 ± 5.53 with no statistical significance in the three different groups, the age-related physiological factors could alter the outcomes. Niroumanesh et al. concluded that older multiparous patients with several previous pregnancies had more tendencies for surgical and curettage approaches than the other patients [24].

Another conclusive point about the patient’s mean age is that younger patients experiencing abortion need to be educated thoroughly about contraceptives and preventive methods, unlike the other age groups. However, this perspective might appear irrelevant to the current study’s aim.

Abnormal weight in mothers has a crucial impact on fertility and sanitizations during a healthy pregnancy; that is why determining factors like Body mass index (BMI) like weight and height can play a substantial role in abortion physiology. All the patients were examined for weight and blood pressure, but no statistical significance in these variables was noted that might have altered the outcomes. These findings were compatible with a study conducted by Ghasemi et al. [29].

After the prescription of drugs in the different groups, bleeding, pain, and spots, considered pregnancy tissue expulsion-associated complications, were carefully evaluated. A persistent postpartum hemorrhage after fetus abortion signifies small fragments of trophoblast and decidua still retained in the uterus after fetal death or RPOC expulsion [30]. Various medical protocols are developed to prevent bleeding after surgical abortion or labor. However, these protocols are continuously modified to achieve better outcomes and higher success rates [31–33]. Even though there is no significant difference regarding the severity of bleeding in the three groups in our study, those given misoprostol plus methylergonovine showed mild bleeding compared to the other groups.

A study was conducted by Whitehouse et al. to compare the efficacy of medical management for bleeding prevention after surgical abortion in over 336 patients with a history of recent abortion. Their study discovered that 72% of the patients used contraceptive drugs to prevent bleeding and 83% preferred vasopressin over other medications. Likewise, they declared that these patients were willing to intake methylergonovine during the second trimester to control severe bleeding and then preferred misoprostol [17]. Another study by Whitehouse et al. illustrated oxytocin’s potency in reducing the frequency rate of bleeding and blood loss. They concluded that oxytocin as prophylaxis for bleeding does not result in a reduced number of interventions required to control the bleeding during the dilation and evacuation (D&E) period between 18 and 24 weeks of gestation. However, it does reduce the bleeding intensity and hemorrhage frequency, which was also in harmony with our study [34].

Methylergonovine and oxytocin are stimulators of uterine contraction that act directly on uterine smooth muscle and peripheral vasculature, especially uterine vessels, and thus reduce bleeding [35, 36].

Similarly, regarding spotting manifestation after abortion and medication intake, the analysis proved no significant difference between different study groups. This fact confirms the results obtained from the degree of bleeding reported by the participants.

Considering medication intake and postpartum pain followed by fetal abortion, our study has discovered that the combination of misoprostol plus methylergonovine showed the best efficacy in pain management. A varied number of guidelines published for pain management by various medical associations have recommended only a normal dosage of common analgesics [37, 38]. However, most of these guidelines describe neither a specific medication type nor a specific dosage in this scope. Moreover, the efficacy of analgesic protocols is limitedly studied in the literature, especially those discussing the prevention and treatment of pain post-miscarriage.

Although they strongly recommend controlling, monitoring, and measuring the pain [39], they have not specified pain measurement methods in women with miscarriages during the first trimester.

A study by Kemppainen et al. [40] on pain scaling during a medical abortion in early pregnancy reported a VAS score. Of all the women, 57.7% experienced severe pain (VAS = 70) during abortion care. Also, 93.5% of these women needed additional opioid analgesics such as tramadol or oxycodone despite being administered with regular analgesics for prophylactic pain medication, like ibuprofen and paracetamol. Even though the misoprostol administration did not reduce the risk of pain recurrence, patients treated with misoprostol reported satisfaction. According to Braaten et al. [41], intravenous injections of sedatives did not help reduce the pain in patients with surgical abortion during the first trimester; in contrast to their study, due to the administration of combined misoprostol and methylergonovine, severe pain remained uncovered, and its manifestation occurred in none of the patients. It is noteworthy to mention that methylergometrine can also be used to modulate the pain of origins except miscarriage; for example, Niño-Maldonado et al. reported positive efficacy of methylergometrine for migraine pain modulation in emergency settings [42].

### Limitations

Our study had several limitations. First, Short-term follow-up and small sample size were among the barriers that limited the present study. Second, in this study, to evaluate bleeding, only the severity of bleeding was considered, and the volume of bleeding and the average number of days during which there is bleeding was not assessed.

## Conclusion

Outpatient treatment in patients with miscarriage can be carried out by administering oxytocin and methylergometrine in combination with misoprostol. This form of medical therapy has positively impacted the expulsion of RPOC during the first trimester and was more cost-effective compared to surgical and hospitalization approaches. Moreover, outpatient treatments in cases of medical abortion and avoidance of hospitalization are advantageous, especially during the COVID-19 pandemic. Not only does it preserve the medical care resources and service management, and effectively improve women’s physical and mental health status, but it is also more convenient for the patients to manage the pain at ease as they receive psychological support from their families at home. Besides, these patients with outpatient treatment often find the opportunity to handle housework affairs and can also meet other children’s needs at home.

## Data Availability

The datasets generated and analyzed during the current research are not publicly available as individual privacy could be compromised, but are available from the corresponding author on reasonable request.

## Abbreviations

WHO: World Health Organization, RPOC:retained products of conception
LMP: Last Menstrual Period
IUD: Intrauterine device, VAS:Visual Analogue Scale system
